# Predictive value of gamma-glutamyltransferase for ventricular arrhythmias and cardiovascular mortality in implantable cardioverter-defibrillator patients

**DOI:** 10.1186/s12872-019-1114-3

**Published:** 2019-05-30

**Authors:** You Zhou, Shuang Zhao, Keping Chen, Wei Hua, Shu Zhang

**Affiliations:** State Key Laboratory of Cardiovascular Disease, Arrhythmia Center, Fuwai Hospital, National Center for Cardiovascular Diseases, Chinese Academy of Medical Sciences and Peking Union Medical College, 167 Bei Li Shi Road, Xicheng District, Beijing, 100037 China

**Keywords:** Gamma-glutamyltransferase, Implantable cardioverter defibrillator, Home monitoring, Ventricular arrhythmias, Cardiac death

## Abstract

**Background:**

Gamma-glutamyltransferase (GGT) is a new predictor of cardiovascular diseases. In this study, we aimed to determine its association with ventricular arrhythmias (VAs) in implantable cardioverter-defibrillator (ICD) patients.

**Methods:**

One hundred and forty patients implanted with ICD or cardiac resynchronization therapy defibrillator with home monitoring were studied retrospectively. The primary endpoint was appropriate ICD treatment of VAs, secondary endpoint was cardiac death.

**Results:**

During a mean follow-up period of 44 ± 17 months, 78 patients (55.7%) experienced VAs, 50 patients (35.7%) were treated with appropriate ICD shocks and 16 patients (11.4%) died due to cardiovascular diseases. GGT was positively correlated with high sensitivity C reactive protein (r = 0.482, *P* < 0.001), left ventricular end-diastolic dimension (r = 0.175, *P* = 0.039), New York Heart Association class (r = 0.199, *P* = 0.018), fasting blood glucose (r = 0.233, *P* = 0.006) and negatively with left ventricular ejection fraction (r = − 0.181, *P* = 0.032) and high-density lipoprotein (r = − 0.313, *P* < 0.001). Based on receiver operating characteristics curve, the cut-off value of GGT = 56 U/L was identified to predict VAs. In Kaplan-Meier survival analysis, GGT ≥56 U/L was associated with increased VAs (P<0.001), ICD shock events (*P* = 0.006) and cardiovascular mortality (*P* = 0.003). In multivariate COX regression models, GGT ≥56 U/L was an independent risk factor for VAs (HR 2.253, 95%CI:1.383–3.671, *P* = 0.001), ICD shocks (HR 2.256, 95%CI:1.219–4.176, *P* = 0.010) and cardiac death (HR 3.555, 95%CI:1.215–10.404, *P* = 0.021).

**Conclusions:**

In this ICD population, GGT ≥56 U/L was independently associated with VAs and cardiac death.

## Background

Sudden cardiac death (SCD) remains a major public health problem accounting for 50% of cardiovascular death despite advances in medical and device therapies [1]. About 80% of SCD are caused by ventricular tachycardia (VT) or ventricular fibrillation (VF) [2]. Since 1980, implantable cardioverter defibrillator (ICD) has been used to identify and terminate malignant ventricular arrhythmias (VAs) to prevent SCD. A low left ventricular ejection fraction (LVEF; < 35%) has been used to predict SCD, but LVEF alone is not enough to fully identify the high-risk population. Many ICD patients never experienced VAs demanding ICD therapy. Therefore, it is important to identify predictors of VAs, permitting timely therapeutic interventions to improve clinical outcomes.

Home Monitoring (HM) technology in ICD patients enables continuous and instantaneous transmission of stored ICD data, which helps to timely and precisely identify the VA episodes. Previous study proved HM with automatic daily surveillance to be safe and effective [3].

Gamma-glutamyltransferase (GGT) plays a key role in the synthesis and metabolism of glutathione. Elevated GGT is a marker of antioxidant inadequacy and increased oxidative stress. Ample evidence suggested that elevated GGT was associated with increased risk of cardiovascular diseases, such as coronary heart disease, heart failure, hypertension, atrial fibrillation and SCD [4–8]. However, its impact on VAs occurrence and survival in ICD patients remains unclear. This study aimed to use ICD home monitoring feature to evaluate the predictive value of GGT for VAs and cardiac death.

## Methods

### Study population

A total of 140 patients who underwent ICD or cardiac resynchronization therapy defibrillator (CRT-D) implantation in Fuwai Hospital between June 2010 and June 2014 and met the inclusion criteria were enrolled in our study. Indications for device implantation in this study were defined in accordance with the published guidelines for device-based therapy [9]. Primary prevention patients were those who received ICDs or CRT-Ds on a prophylactic basis without a prior history of SCD, cardiac arrest, or sustained VT. Secondary prevention patients were those who experienced resuscitated SCD, cardiac arrest, or sustained VT before ICD implantation. The study was approved by the hospital ethics committee, and all patients provided written informed consent before entering this study.

### Selection criteria

The inclusion criteria were (1) patients with ICD/CRT-D devices (Biotronik, Berlin, Germany) equipped with HM that could process daily HM transmissions, and (2) patients with GGT evaluations on admission.

### Demographic and clinical characteristics

Patients’ demographic characteristics including age, gender and body mass index, and baseline clinical characteristics, including echocardiographic parameters, New York Heart Association (NYHA) class, comorbidities (ischemic cardiomyopathy, hypertension, atrial fibrillation, diabetes), laboratory variables, including GGT, high-sensitivity C-reactive protein (hsCRP), high density lipoprotein (HDL), low density lipoprotein (LDL), triglyceride, fasting blood glucose (FBG), and medications (beta-blockers, amiodarone, diuretics and angiotensin-converting enzyme inhibitors or angiotensin receptor blockers) were obtained from patients’ medical records prior to device implantation.

### Biochemical analyses

Venous blood samples after 12 h fasting were collected from each patient on the day before device implantation. All these tests were measured in the core laboratory of Fuwai Hospital by standard techniques. GGT and other biochemical parameters, including creatinine, FBG, HDL, LDL, triglyceride, were determined by Hitachi 7180 biochemistry autoanalyzer. The concentrations of hsCRP were examined using immunoturbidimetry (Beckmann Assay 360, Bera, CA, USA). The Cockcroft-Gault equation was used to estimate glomerular filtration rate.

### Device settings

The ICD programmed settings were as follows: the basic pacing rate was 40–60 bpm, VT monitor zone was 140–170 bpm, VT therapy zone was 170–210 bpm, and VF zone was over 210 bpm. All devices were programmed to provide continuous patient monitoring data.

### Endpoints

The primary endpoint was the appropriate ICD therapy for VA and the secondary endpoint was cardiac death. VA was identified from the archived HM data and confirmed by intracardiac electrograms. Inappropriate events were excluded. Routine follow-ups were conducted and the status of the patient was confirmed by telephone if the patient’s transmission was disrupted. If a patient died, the date and cause of death were confirmed by contacting the family.

### Statistical analysis

Continuous variables were presented as the means (± SDs) or medians (inter-quartile range). Student’s t tests and Mann-Whitney U tests were used to compare normally and non-normally distributed variables, respectively. Categorical variables in each group were presented as percentages and were compared by the χ ^2^ test. Spearman test was used to evaluate the correlation between GGT and other clinical or laboratory variables. Receiver operating characteristic (ROC) curve was plotted to identify a GGT cut-off value that could be used to predict VAs. The Kaplan-Meier method was used to draw the survival rate curves and the log-rank test was used to compare the differences between the curves. Multiple Cox regression analyses were performed with an enter regression model in which age, gender and each variable with a *P* value < 0.05 (based on the univariate analysis) were entered into the model. A P value < 0.05 was considered statistically significant. SPSS Statistics 23.0 (SPSS, Chicago, IL, USA) and Graph Pad Prism Software 6.0 (GraphPad Software, La Jolla, CA, USA) were used to perform the statistics.

## Results

### Baseline characteristics

A total of 140 patients were included into this study. The mean follow-up period was 44 ± 17 months. Men were dominant in the study cohort (75.0%). The average age was 56.2 ± 13.1 years. During the follow-ups, 78 patients (55.7%) experienced VAs, 50 patients (35.7%) received ICD shock therapy, and 16 patients (11.4%) died of cardiovascular diseases.

The mean GGT on admission was 50.4 ± 46.1 U/L. ROC curve analysis determined that a GGT cut-off value of 56 U/L could predict VA. The area under the curve was 0.635 (95% CI:0.543–0.727, *P* = 0.006) with a sensitivity of 43.6% and a specificity of 87.1%. Comparisons of patients’ baseline characteristics grouped according to the GGT cut-off value of 56 U/L were presented in Table 1. Patients whose GGT ≥56 U/L were more likely to be male and receive diuretics and spironolactone, with lower LVEF and higher hsCRP and FBG, than patients whose GGT < 56 U/L.

**Table 1:** Baseline characteristics according to GGT

	Total population (*n* = 140)	GGT < 56 U/L (*n* = 98)	GGT ≥56 U/L (*n* = 42)	P value
Age (years)	56.2 ± 13.1	56.1 ± 13.0	56.5 ± 13.4	0.540
Male	105 (75.0%)	65 (66.3%)	40 (95.2%)	< 0.001
BMI (Kg·m^− 2^)	24.2 ± 2.4	24.2 ± 2.2	24.4 ± 2.7	0.858
Primary prevention	25 (17.9%)	16 (16.3%)	9 (21.4%)	0.470
Ischemic cardiomyopathy	36 (25.7%)	22 (22.4%)	14 (33.3%)	0.177
Hypertension	31 (22.1%)	24 (24.5%)	7 (16.7%)	0.307
Diabetes	4 (2.9%)	3 (3.1%)	1 (2.4%)	0.825
Atrial fibrillation	10 (7.1%)	5 (5.1%)	5 (11.9%)	0.152
QRS duration	112.49 ± 26.89	111.40 ± 26.27	115.72 ± 29.19	0.559
LVEF (%)	44.9 ± 14.7	46.6 ± 14.6	40.9 ± 14.4	0.034
LVEDD (mm)	57.1 ± 13.3	55.9 ± 13.0	60.0 ± 13.7	0.094
NYHA class I-II	95 (67.9%)	71 (72.5%)	24 (57.1%)	0.077
ACEI or ARB	74 (52.9%)	57 (58.2%)	17 (40.5%)	0.055
Beta-blocker	122 (87.1%)	86 (87.6%)	36 (85.7%)	0.741
Amiodarone	80 (57.1%)	56 (57.1%)	24 (57.1%)	1.000
Spironolactone	76 (54.3%)	44 (44.9%)	32 (76.2%)	0.001
Digoxin	38 (27.1%)	26 (26.5%)	12 (28.6%)	0.803
Diuretic	84 (60.0%)	53 (54.1%)	31 (73.8%)	0.029
Statins	50 (35.7%)	39 (39.8%)	11 (26.2%)	0.124
hsCRP (mg·L^−1^)	4.61 ± 4.74	3.58 ± 4.06	7.03 ± 5.42	< 0.001
eGFR (mL·min^−1^·1.73 m^−2^)	80.0 ± 24.5	81.8 ± 24.8	75.8 ± 23.5	0.189
FBG (mmol·L^−1^)	5.34 ± 1.20	5.14 ± 1.00	5.80 ± 1.50	0.011
HDL (mmol·L^−1^)	1.03 ± 0.36	1.13 ± 0.31	1.07 ± 0.47	0.365
LDL (mmol·L^−1^)	2.45 ± 0.73	2.44 ± 0.74	2.47 ± 0.74	0.849
Triglyceride (mmol·L^−1^)	1.51 ± 0.84	1.51 ± 0.89	1.53 ± 0.74	0.514

Values are expressed as the mean ± SD or n (%)

Abbreviations: *BMI* body mass index, *LVEF* left ventricular ejection fraction, *LVEDD* left ventricular end-diastolic dimension, NYHA class, New York Heart Association class, *ACEI or ARB* angiotensin-converting enzyme inhibitor or angiotensin receptor blocker, *hsCRP* high-sensitivity C-reactive protein, *eGFR* estimated glomerular filtration rate, *FBG* fasting blood glucose, *HDL* high density lipoprotein, *LDL* low density lipoprotein, *GGT* gamma-glutamyltransferase

### The relationship between GGT and baseline variables

In correlation analysis, GGT was positively correlated with hsCRP (r = 0.482, *P* < 0.001), left ventricular end-diastolic dimension (LVEDD; r = 0.175, *P* = 0.039), NYHA class (r = 0.199, *P* = 0.018), FBG (r = 0.233, *P* = 0.006) and negatively with LVEF (r = − 0.181, *P* = 0.032) and HDL (r = − 0.313, P < 0.001) (Table 2).

**Table 2:** Correlation analysis between GGT and Baseline Variables

Baseline Variables	r	*P*-value
Body mass index	0.094	0.273
QRS duration	0.191	0.111
Left ventricular ejection fraction	−0.181	0.032
Left ventricular end-diastolic dimension	0.175	0.039
New York Heart Association class	0.199	0.018
High-sensitivity C-reactive protein	0.482	<0.001
Estimated glomerular filtration rate	−0.140	0.099
Fasting blood glucose	0.233	0.006
High density lipoprotein	−0.313	<0.001
Low density lipoprotein	0.035	0.682
Triglyceride	0.100	0.241

Abbreviation: *GGT* gamma-glutamyltransferase

### GGT was a predictor of VAs, shock events and cardiac death

**Fig. 1:**
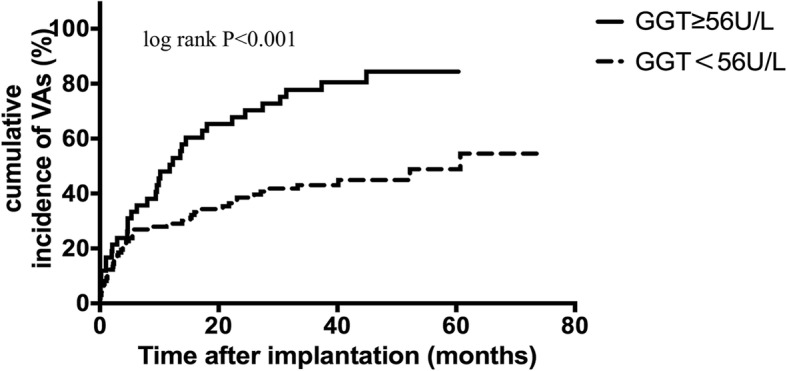
Kaplan-Meier estimates of the cumulative incidence of VAs (log rank *P* < 0.001). Abbreviations: VAs, ventricular arrhythmias

**Fig. 2:**
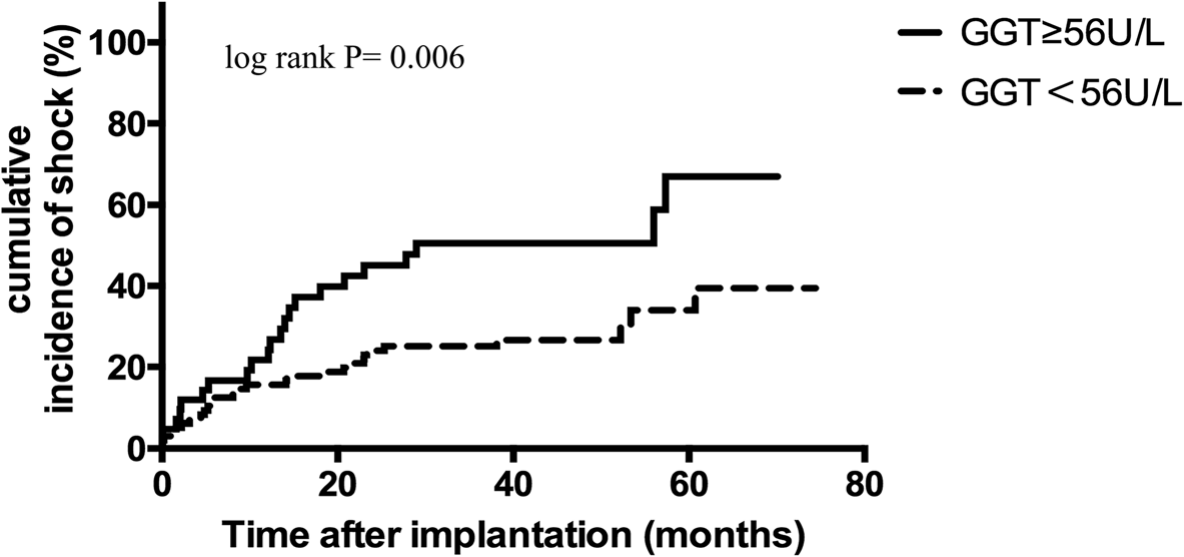
Kaplan-Meier estimates of the cumulative incidence of shocks (log rank *P* = 0.006)

**Fig. 3:**
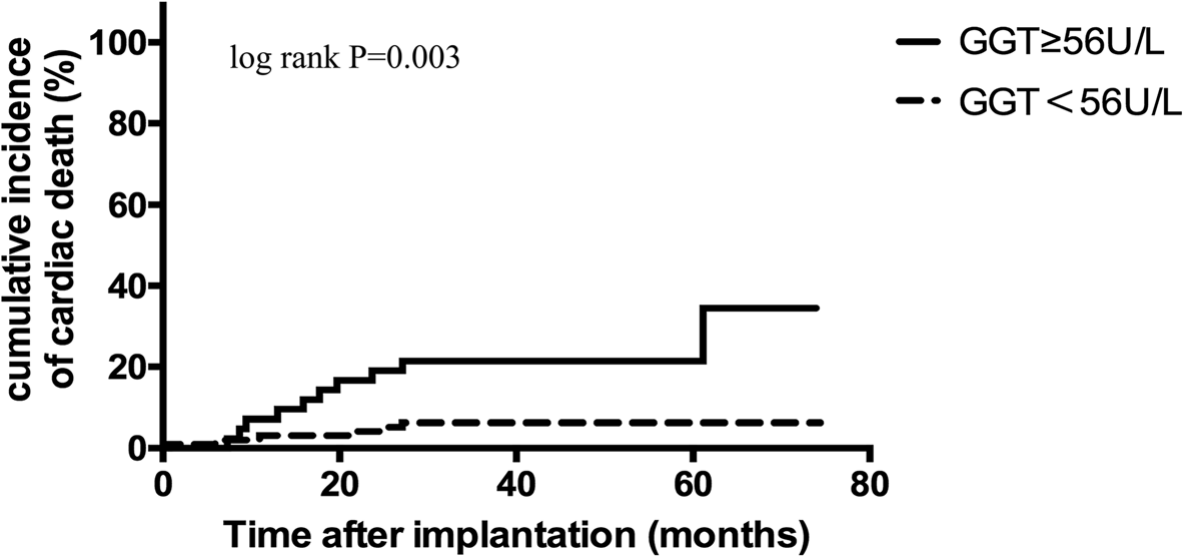
Kaplan-Meier estimates of the cumulative incidence of cardiac death (log rank *P* = 0.003)

Kaplan-Meier survival curves showed a higher incidence of VAs, shock therapy and cardiac death in patients with GGT ≥56 U/L (P = < 0.001, 0.006, 0.003, respectively; Figs. 1, 2 and 3, respectively). Adjusted by age, gender and other variables with *P* value < 0.05 in the univariate analysis, multivariate Cox regression analysis revealed that GGT ≥56 U/L was an independent predictor for VA (HR 2.253, 95%CI:1.383–3.671, *P* = 0.001; adjusted for age, gender, LVEDD, ischemic cardiomyopathy and diabetes), ICD shock therapy (HR 2.256, 95%CI:1.219–4.176, *P* = 0.010; adjusted for age, gender, LVEDD and ischemic cardiomyopathy) and cardiac death (HR 3.555, 95%CI:1.215–10.404, *P* = 0.021; adjusted for age, gender, LVEDD, LVEF and diabetes) (Table 3).

**Table 3:** Univariate and multivariate Cox analysis for endpoints

	HR	95%CI	P value
univariate analysis			
VAs			
GGT ≥ 56 U/L	2.429	1.542–3.826	<0.001
shock			
GGT ≥ 56 U/L	2.161	1.234–3.784	0.007
cardiac death			
GGT ≥ 56 U/L	4.171	1.515–11.483	0.006
multivariate analysis			
VAs^a^			
GGT ≥ 56 U/L	2.253	1.383–3.671	0.001
Diabetes	3.168	1.082–9.276	0.035
shock^b^			
GGT ≥ 56 U/L	2.256	1.219–4.176	0.010
LVEDD	1.026	1.003–1.050	0.029
cardiac death^c^			
GGT ≥ 56 U/L	3.555	1.215–10.404	0.021
Diabetes	5.725	1.191–27.515	0.029

a adjusted for age, gender, LVEDD, ischemic cardiomyopathy, diabetes, GGT ≥ 56 U/L

b adjusted for age, gender, LVEDD, ischemic cardiomyopathy, GGT ≥ 56 U/L

c adjusted for age, gender, LVEDD, left ventricular ejection fraction, diabetes, GGT ≥ 56 U/L

Abbreviations: *VAs* ventricular arrhythmias, *HR* hazard ratio, *CI* confidence interval, *GGT* gamma-glutamyltransferase, *LVEDD* left ventricular end-diastolic diameter

## Discussion

This study showed that GGT ≥56 U/L was an independent risk factor for VA, ICD shock therapy and cardiovascular death in ICD patients. GGT is a key enzyme in the extracellular metabolism of glutathione and is considered as a marker of oxidative stress and inflammation [10]. GGT may also be directly involved in the pathophysiology of atherosclerosis in view of the presence of catalytically active GGT in atherosclerotic plaques and a correlation between GGT activity and plaque instability [11, 12]. Many studies demonstrated that elevated GGT was associated with increased risk of cardiovascular diseases. A meta-analysis including over 1.23 million subjects showed a positive association in a log-linear fashion between elevated GGT and cardiovascular diseases (RR = 1.23[1.16–1.29]; *P* < 0.001) [4]. Poelzl et al.’s study demonstrated that GGT was associated with disease severity and an independent predictor of death or heart transplantation in patients with chronic heart failure [5]. Some studies also supported that elevated GGT was associated with VA. A cohort study of 1780 Finnish men demonstrated that GGT activity was positively and log-linearly associated with future risk of VA and SCD [8, 13]. Elevated GGT was also found to be an independent risk factor for VT in patients with type 2 diabetes [14]. However, these studies focused on community population or patients without high SCD risk. The patients included in our study were at high risk for SCD and most had structural heart disease with decreased LVEF. GGT could predict life-threatening VA in this population. On the other hand, racial disparities may also affect the predictive power of GGT [15]. This study proved predictive value of GGT in Asian population with high SCD risk.

Oxidative stress and inflammation may play a role between GGT and arrhythmogenesis. Oxidative stress can lead to VA or SCD by inducing myocardial ischemia, remodeling of ion channels and affecting autonomic nervous system function [16]. Parajuli et al. [17] found that, in the heart specimens of patients with non-ischemic cardiomyopathy, patients with VA than those without VA had a reduction in the ratio of glutathione and oxidized glutathione with an increase in NADPH oxidase activity, accompanied by increased TRPM7 expression and Connexin 43 redistribution. Inflammatory cytokines such as tumor necrosis factor -α and interleukin − 6 had also been found involved in the development of VA by modulating ion channels [18]. Anti-inflammatory therapy was proved to reduce spontaneous and inducible VAs in murine models [19]. Our study found a moderate positive correlation between GGT and hsCRP, and patients in GGT ≥56 U / L group had significantly higher hsCRP, indicating elevated GGT was associated with systemic inflammation. Lee et al. [20] also found that GGT and CRP were positively correlated and GGT could be raised before elevation of serum CRP concentration. Oxidative stress consumes glutathione, so elevated GGT may be its compensatory response. Oxidative stress is also closely related to carbohydrate and lipid metabolism. In the present study, GGT was correlated with FBG and HDL, and diabetes mellitus was also found to be an independent predictor of VA. In patients with new-onset diabetes and pre-diabetes, elevated GGT was associated with prolonged QT interval and increased QT dispersion [21]. Elevated GGT is associated with oxidative stress and systemic inflammation, thus reflecting electrophysiological instability. Compared with GGT < 56 U/L group, patients with GGT ≥56 U/L had lower LVEF and more patients used diuretics and spironolactone to treat heart failure. The correlation analysis also showed that GGT was negatively correlated with LVEF and positively correlated with LVEDD and NYHA class. The possible reason might be hepatic congestion caused by left ventricular dysfunction, and antioxidant inadequacy and increased oxidative stress, which caused elevated GGT and involved in ventricular remodeling. The association between GGT and NYHA class, LVEF and LVEDD were also found in previous studies [4, 22]. Patients with more advanced heart failure might need diuretics and spironolactone to further control syndrome. In Poelzl et al.’s study, heart failure patients with elevated GGT were also more on diuretics and spironolactone [5]. Thus, heart function might also play a role in GGT for predicting VA.

The association between GGT and cardiac death had been found in a large number of studies. Framingham offspring study found that GGT could be used as a marker for metabolic syndrome and cardiovascular diseases and predict the risk of death [23]. In a 17-year follow-up, an Austrian cohort study of 163,944 adults revealed higher GGT was significantly associated with cardiovascular mortality, showing a clear dose-response relationship [24]. A meta-analysis by Wang et al. [25] also confirmed a positive correlation between GGT and the risk of cardiovascular death. In our study, GGT ≥56 U / L was associated with 3.5-fold risk of cardiovascular death in ICD patients, which proved GGT’s predictive value in ICD patients. GGT might be helpful in risk stratification in this population.

## Study limitations

This retrospective single-center study had a relatively small sample size and included a mixture of patients receiving ICDs or CRT-Ds for primary or secondary prevention. Predictive values of GGT could decrease in female or the elderly [26, 27]. However, further subgroup analysis according to gender or age did not make sense for statistical reasons. We also did not measure GGT during follow-up to observe its change. Therefore, a prospective study that includes adequate number of patients is necessary to confirm the prognostic value of GGT. Secondly, racial disparities also affected the predictive power of GGT [15]. This study only included Chinese patients. The results may not apply to other ethnic populations. Finally, only one inflammatory biomarker (hsCRP) was analyzed and compared to GGT. Other inflammatory cytokines such as tumor necrosis factor -α and interleukin − 6 were not available in this retrospective study.

## Conclusions

To conclude, elevated GGT is associated with VAs and cardiac death in ICD patients. GGT measurements may have the potential to improve patient selection for ICD therapy. Further studies are warranted to verify our findings.

## Data Availability

The datasets generated and analysed during the current study are not publicly available due to the Fuwai Hospital regulations, but are available from the corresponding author on reasonable request.

## Abbreviations

CRT-D: Cardiac resynchronization therapy defibrillator
FBG: Fasting blood glucose
GGT: Gamma-glutamyltransferase
HDL: High density lipoprotein
HM: Home monitoring
hsCRP: High-sensitivity C-reactive protein,
ICD: Implantable cardioverter defibrillator
LDL: Low density lipoprotein
LVEDD: Left ventricular end-diastolic dimension
LVEF: Left ventricular ejection fraction
NYHA: New York heart association
ROC: Receiver operating characteristic
SCD: Sudden cardiac death
VA: Ventricular arrhythmias
VF: Ventricular fibrillation
VT: Ventricular tachycardia
